# Targeting the Leukemic stem cell protein machinery by inhibition of mitochondrial pyrimidine synthesis

**DOI:** 10.15252/emmm.202216171

**Published:** 2022-06-13

**Authors:** Elisa Donato, Andreas Trumpp

**Affiliations:** ^1^ Division of Stem Cells and Cancer German Cancer Research Center (DKFZ) and DKFZ‐ZMBH Alliance Heidelberg Germany; ^2^ Heidelberg Institute for Stem Cell Technology and Experimental Medicine (HI‐STEM gGmbH) Heidelberg Germany

## Abstract

Acute Myeloid Leukemia is one of the most aggressive blood cancers with a high frequency of relapse. While standard chemotherapy is able to target rapidly proliferating immature blasts, it fails to eradicate slowly proliferating Leukemic Stem Cells. Therefore, new therapeutic strategies that efficiently target LSCs are urgently needed. Recent studies suggest that LSCs have particular metabolic vulnerabilities, which would open the possibility of a therapeutic window with limited off‐target effects on the normal hematopoietic system. In this issue of EMBO Molecular Medicine, So and colleagues investigate the mechanism of action of AG636, a new potent inhibitor of *de novo* pyrimidine synthesis, and discovered an unexpected link to AML protein translation essential for LSC function.

## Graphical abstract

E. Donato and A. Trumpp discuss the study by So *et al* (in this issue of EMBO Mol Med) that investigates the mechanism of action of AG636, a new potent inhibitor of de novo pyrimidine synthesis, in the context of acute myeloid leukemia.

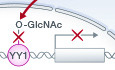

AML is the most aggressive blood cancer with a 5‐year survival rate lower than 20% for patients older than 65 years. While the majority of patients initially reach complete remission after standard chemotherapy, the rate of mortality remains very high due to recurrent disease. The formation of AML relapse is ascribed to the persistence of chemotherapy‐resistant LSCs with long‐term self‐renewal activity able to regenerate the disease. Previous studies have demonstrated that AMLs show a hierarchical organization with quiescent/low cycling LSCs at the top of the tree giving rise to fast proliferating immature myeloid blasts unable to generate terminally differentiated mature cells (Trumpp & Haas, 2022). While standard chemotherapy with high doses of Cytarabine combined with Anthracyclines successfully eradicates proliferating immature blasts, in most of the cases, these regimens leave at least some LSCs untouched. Thus, there is an unmet need to develop strategies to efficiently eradicate LSCs in first‐line therapies with the goal to prevent secondary relapse.

There is increasing evidence that AML cells as well as LSCs exhibit metabolic vulnerabilities. In particular, differences between healthy hematopoietic and leukemic systems have been identified, offering promise for potential therapeutic windows.

Healthy Hematopoietic Stem Cells are quiescent cells likely residing in a hypoxic niche using glycolysis for energy production (Simsek *et al*, 2010). Simultaneously, the limited mitochondrial respiration keeps ROS levels low, thus contributing to the maintenance of genomic and mitochondrial DNA integrity of HSCs (Walter *et al*, 2015). During expansion and differentiation, progenitors switch to OxPHOS to sustain the higher energy demand. Similar to HSCs, also LSCs display low levels of mitochondrial respiration and ROS compared to leukemia blasts. Nonetheless, LSCs do still rely on OxPHOS for energy production (Lagadinou *et al*, 2013). Indeed, despite their higher mitochondrial mass, AML blasts and LSCs have low spare reserve capacity, being therefore more dependent on OxPHOS and more susceptible to oxidative stress compared to their healthy counterparts (Skrtic *et al*, 2011). Finally, LSCs can use fatty acids to fuel the TCA cycle and OxPHOS. In fact, chemo‐resistant AML cells can upregulate CD36, a fatty acid transporter, to increase the import of fatty acid released by the adipocytes in the extracellular niche (Farge *et al*, 2017). The increasing understanding of AML (and especially LSCs) metabolic vulnerabilities are promoting new clinical trials targeting mitochondrial functions. A successful example is the BCL‐2 inhibitor Venetoclax already introduced in clinical practice (de Beauchamp *et al*, 2022).

An alternative approach to target mitochondrial function is inhibition of Dihydroorotate dehydrogenase (DHODH), an essential enzyme localized in the mitochondrial inner membrane and catalyzing the fourth step of the *de novo* pyrimidine synthesis. DHODH links nucleotide synthesis with energy metabolism and ROS production. It was recently demonstrated that inhibition of DHODH, associated with consequent pyrimidine starvation, leads to differentiation and/or death of AML blasts (Sykes *et al*, 2016). In 2020, the Scadden laboratory showed that chemotherapy‐persisting cells are dependent on pyrimidine synthesis and that DHODH inhibition was significantly reducing tumor burden *in vivo* when used in combination with standard chemotherapy (van Gastel *et al*, 2020). This link between pyrimidine starvation and blast differentiation generated an increasing effort in developing novel and potent DHODH inhibitors usable to target AML stem cells in patients.

In this issue of EMBO Molecular Medicine, So and colleagues report findings from a preclinical study assessing the novel potent DHODH inhibitor AG636 (So *et al*, 2022). They investigated the molecular mechanism behind AML blast differentiation and LSCs clearance in response to AG636 treatment. Using an MLL rearranged AML mouse model, they showed that one cycle of AG636 treatment was sufficient to induce blast differentiation and LSCs reduction, while with four treatment cycles complete remission was achieved with no detectable leukemic cells in the blood, thus presumably also eliminating LSCs. The central role of the *de novo* pyrimidine synthesis is not limited to MLL rearranged AMLs, since also AML1‐ETO or IDH1^R132H^/DNMT3A^R882H^/Nras^G12D^ mouse models were susceptible to DHODH inhibition. Even though AG636 treatment was associated with reduction of normal B cells, myeloid and megakaryocyte‐erythroid progenitors as well as LT‐HSCs, this effect was transient and normal hematopoiesis returned to baseline after drug discontinuation. Transcriptomic analyses of enriched LSCs (cKit^high^ CD11b^low^) identify genes associated with protein synthesis as strongly deregulated after DHODH inhibition. This observation was further validated by polysome profiling and L‐Azidohomoalanine incorporation, functionally demonstrating that AG636 treatment leads to the inhibition of the protein translation machinery. By chromatin accessibility analysis on enriched LSCs (cKit^high^ CD11b^low^) and data mining using publically available datasets, the authors identified YY1 as a potential transcriptional regulator of genes controlling protein translation. This observation was particularly interesting since YY1 is post‐translationally modified by the addition of O‐GlcNAc: a post‐translational modification dependent on pyrimidine biosynthesis. The crucial role of O‐GlcNAc modification was confirmed by rescue experiments with an inhibitor of O‐GlcNAcase (PUGNAc). Thus, *de novo* pyrimidine synthesis inhibition by AG636 is altering YY1 stability by preventing O‐GlcNAc modification with consequent downregulation of its target genes (Fig 1).

**Figure 1:**
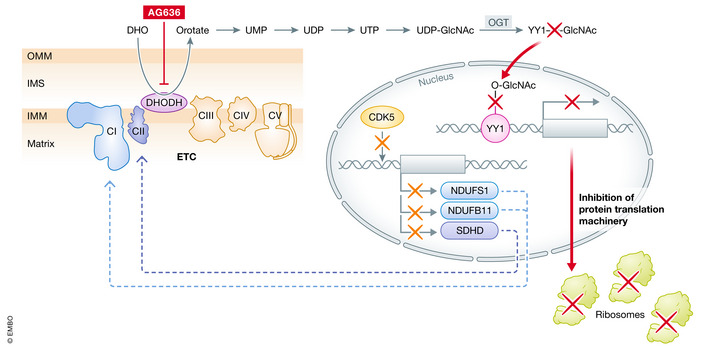
Schematic representation of cellular response to DHODH inhibition DHODH catalyzes the conversion of DHO to orotate by transferring electrons from coenzyme Q10. When pyrimidines are available, OGT adds O‐GlcNAc to newly synthesized proteins. YY1 is modified by OGT and is involved in the transcriptional control of genes regulating protein synthesis. When DHODH is inhibited by AG636, pyrimidines are not synthetized and OGT cannot add O‐GlcNAc residues to target proteins. This leads to a reduction in ribosome assembly and protein translation. CDK5 is involved in the transcription of components of mitochondrial ETC complex I and III.

Finally, by performing CRISPR‐Cas9 KO screens using a focused library of epigenetic regulators, the authors identified CDK5 and members of the INO80 chromatin remodeling complex as sensitizer to DHODH inhibition. Transcriptomic analysis of CDK5 knockout cells treated with AG636 revealed numerous genes involved in OxPHOS and the Electron Transport Chain as being strongly downregulated, suggesting that the synthetic lethality could be explained by a dual synergistic attack on mitochondria (Fig 1).

In summary, So and colleagues shed new light on the mechanism of action of DHODH inhibition, further proving LSC and blasts dependency on *de novo* pyrimidine synthesis for survival and cell fate decision, respectively. Interestingly, in LSCs, pyrimidine starvation is controlling protein modification and stability of key transcription regulators and YY1 was identified as a novel regulator of protein translation in AML. Finally, synthetic lethality was observed when DHODH inhibition was concomitant with depletion of CDK5. Indeed, the authors showed for the first time that CDK5 regulates the expression of ETC complex I and II components (Fig 1). These findings highlight the importance of simultaneous targeting of different mitochondrial processes, observations that are relevant for the design of future clinical trials. It has to be considered that beside YY1 also other well‐known players in tumor biology such as MYC and DOT1L are post‐translationally modified by the addition of O‐GlcNAc. In addition, MYC also promotes ribosome biogenesis and protein translation. Therefore, a more complex mechanism in which YY1 may only be partially responsible for the cellular effects of DHODH inhibition remains possible. The important work by So *et al* will certainly guide future investigations dissecting the full spectrum of the roles of pyrimidines in AML.

## References

[emmm202216171-bib-0001] de Beauchamp L , Himonas E , Helgason GV (2022) Mitochondrial metabolism as a potential therapeutic target in myeloid leukaemia. Leukemia 36: 1–12 34561557 10.1038/s41375-021-01416-w PMC8727299

[emmm202216171-bib-0002] Farge T , Saland E , de Toni F , Aroua N , Hosseini M , Perry R , Bosc C , Sugita M , Stuani L , Fraisse M *et al* (2017) Chemotherapy‐resistant human acute myeloid leukemia cells are not enriched for leukemic stem cells but require oxidative metabolism. Cancer Discov 7: 716–735 28416471 10.1158/2159-8290.CD-16-0441 PMC5501738

[emmm202216171-bib-0003] van Gastel N , Spinelli JB , Sharda A , Schajnovitz A , Baryawno N , Rhee C , Oki T , Grace E , Soled HJ , Milosevic J *et al* (2020) Induction of a timed metabolic collapse to overcome cancer chemoresistance. Cell Metab 32: 391–403.e396 32763164 10.1016/j.cmet.2020.07.009 PMC8397232

[emmm202216171-bib-0004] Lagadinou E , Sach A , Callahan K , Rossi R , Neering S , Minhajuddin M , Ashton J , Pei S , Grose V , O’Dwyer K *et al* (2013) BCL‐2 inhibition targets oxidative phosphorylation and selectively eradicates quiescent human leukemia stem cells. Cell Stem Cell 12: 329–341 23333149 10.1016/j.stem.2012.12.013 PMC3595363

[emmm202216171-bib-0005] Simsek T , Kocabas F , Zheng J , Deberardinis RJ , Mahmoud AI , Olson EN , Schneider JW , Zhang CC , Sadek HA (2010) The distinct metabolic profile of hematopoietic stem cells reflects their location in a hypoxic niche. Cell Stem Cell 7: 380–390 20804973 10.1016/j.stem.2010.07.011 PMC4159713

[emmm202216171-bib-0006] Škrtić M , Sriskanthadevan S , Jhas B , Gebbia M , Wang X , Wang Z , Hurren R , Jitkova Y , Gronda M , Maclean N *et al* (2011) Inhibition of mitochondrial translation as a therapeutic strategy for human acute myeloid leukemia. Cancer Cell 20: 674–688 22094260 10.1016/j.ccr.2011.10.015 PMC3221282

[emmm202216171-bib-0007] So J , Lewis AC , Smith LK , Stanley K , Franich R , Yoannidis D , Pijpers L , Dominguez P , Hogg S , Vervoort SJ *et al* (2022) Inhibition of pyrimidine biosynthesis targets protein translation in AML. EMBO Mol Med 14: e15203 10.15252/emmm.202115203 PMC9260210 35514210

[emmm202216171-bib-0008] Sykes DB , Kfoury YS , Mercier FE , Wawer MJ , Law JM , Haynes MK , Lewis TA , Schajnovitz A , Jain E , Lee D *et al* (2016) Inhibition of dihydroorotate dehydrogenase overcomes differentiation blockade in acute myeloid leukemia. Cell 167: 171–186.e115 27641501 10.1016/j.cell.2016.08.057 PMC7360335

[emmm202216171-bib-0009] Trumpp A , Haas S (2022) Cancer stem cells: the adventurous journey from hematopoietic to leukemic stem cells. Cell 185: 1266–1270 35385684 10.1016/j.cell.2022.03.025

[emmm202216171-bib-0010] Walter D , Lier A , Geiselhart A , Thalheimer FB , Huntscha S , Sobotta MC , Moehrle B , Brocks D , Bayindir I , Kaschutnig P *et al* (2015) Exit from dormancy provokes DNA‐damage‐induced attrition in haematopoietic stem cells. Nature 520: 549–552 25707806 10.1038/nature14131

